# Evaluation of the PEG Density in the PEGylated Chitosan Nanoparticles as a Drug Carrier for Curcumin and Mitoxantrone

**DOI:** 10.3390/nano8070486

**Published:** 2018-07-01

**Authors:** Yao Chen, Di Wu, Wu Zhong, Shuwen Kuang, Qian Luo, Liujiang Song, Lihua He, Xing Feng, Xiaojun Tao

**Affiliations:** Key Laboratory of Study and Discovery of Small Targeted Molecules of Hunan Province, School of Medicine, Hunan Normal University, Changsha 410013, China; ychen@smail.hunnu.edu.cn (Y.C.); iswudi@smail.hunnu.edu.cn (D.W.); zwu123@smail.hunnu.edu.cn (W.Z.); shwkuang@smail.hunnu.edu.cn (S.K.); luoqian1996@smail.hunnu.edu.cn (Q.L.); song1221@hunnu.edu.cn (L.S.); hlh197@hunnu.edu.cn (L.H.); fengxing@hunnu.edu.cn (X.F.)

**Keywords:** curcumin, mitoxantrone, synergism, PEG, chitosan nanoparticles

## Abstract

Polyethylene glycolated (PEGylated)curcumin-grafted-chitosan (PCC) conjugates were synthesized with three PEG/chitosan feed molar ratios (1/5, 1/7.5, and 1/10), namely PCC1, PCC2 and PCC3. Chemical structures of these conjugates were characterized by Fourier transform infrared (FTIR) and proton nuclear magnetic resonance (^1^H NMR). The degrees of substitution (DS) of PEG were 0.75%, 0.45% and 0.33%, respectively, for PCC1, PCC2 and PCC3by ^1^H NMR analysis. Self-assembled PCC nanoparticles (NPs) were spherical as observed in transmission electron microscope images. Mitoxantrone (MTO)-loaded PCC NPs were prepared to analyze the particle size, zeta potential, drug loading, drug release and in vitro cytotoxicity. The MTO-loaded PCC3 NP (DS = 0.33%) possessed the smallest size (~183.1 nm), highest zeta potential (~+34.0 mV) and the largest loading capacity of curcumin (CUR, ~16.1%) and MTO (~8.30%). The release results showed that MTO-loaded PCC3 NP demonstrated the lowest percentage of MTO release and increased as pH decreased, but the CUR release could only be detected at pH 4.0. In the cytotoxicity study, MTO-loaded PCC3 NP displayed the highest cytotoxicity in HepG2 cell line and the best synergistic effect among the tested NPs. Our results suggest that the DS of PEG has impacts on the structures and functions of PCC NPs: the smaller DS of PEG was associated with the smaller size, the higher zeta potential, the slower drug release, and the higher cytotoxicity of NPs.

## 1. Introduction

Chemotherapy is one of the main strategies for the treatment of late stage of malignant tumors. Curcumin (CUR), a hydrophobic pigment derived from *Curcuma longa*, has been proposed as a good candidate for adjuvant therapy because of its anti-cancer effects, reversal of cancer cell multidrug resistance, and absence of obvious cytotoxicity in normal tissues [1,2,3]. However, clinical application of CUR is limited due to its low water solubility and physico-chemical stability [4]. Recently, the combination chemotherapy of CUR and other drugs has attracted increasing attention on improving therapeutic efficacy by signaling different pathways, and suppressing and reversing drug resistance [3,5,6]. Mitoxantrone (MTO), a chemotherapeutic drug, has curative effect on variety of malignant tumors, but its associated cardiac toxicity and myelosuppression create significant health impairments [7,8]. Studies have shown that the combination of MTO and CUR can enhance the MTO efficacy, therefore, may reduce the MTO dose and the side effects [5,9].

Polymeric nanoparticles have been widely used for biomedical applications especially for drug delivery, which can be formed by self-assembly of amphiphilic polymers in water [10]. Use of polymeric nanoparticles for drug delivery provides many advantages for cancer treatment, including increased drug solubility and stability, enhanced permeability and retention (EPR) effect, easy surface modification and environmental stimuli-responsive properties [11,12]. 

Chitosan (CS) has often been used as the backbone of polymeric nanoparticles because of its basic properties such as good biocompatibility, biodegradability and low toxicity [13,14,15]. Chitosan nanoparticles (CS NPs) with positive surface charges bind to the negatively charged cell membrane, facilitating cellular uptake [16]. However, CS NPs can be recognized and internalized by macrophages in the reticuloendothelial system (RES) [17,18]. Methoxy polyethylene glycol (PEG) was applied to modify CS as a drug carrier [19,20]. PEG was grafted to CS to block the positive charge on the surface of CS NPs so that the CS NPs may escape phagocytosis of RES cells, prolonging their retention time in blood circulation [21]. The length of PEG with the different Mw can affect the size of PEGylated chitosan nanoparticles and the drug delivery functions [20]. We used 2000 Mw PEG and varied the ratio of PEG grafted to evaluate the characteristics and the biological functions of NPs.

In this study, we designed an amphiphilic conjugate by conjugating CUR with CS as hydrophobic moieties and grafting PEG. In aqueous solution, the amphiphilic conjugate automatically forms water-soluble PEGylated CUR-grafted-CS (PCC) NPs. As MTO is encapsulated, it forms the MTO-loaded PCC NPs (PCCM NPs). 

The formation of PCC NPs involves a self-assembly process driven by the direct force of interaction between hydrophobic components and the influence of outer fields such as the template on the surface of NPs. As a result of all factors integrated, the NPs are stabilized under thermodynamic equilibrium [22,23]. We have demonstrated the effects of hydrophobicity in formation of NPs using different degrees of substitution (DS) of cholesterol in pullulan nanoparticles and have reported a method for connecting a hydrophobic molecule to produce an amphiphilic conjugate [24,25]. Excessive substitution of hydrophobic molecules in the core would result in NPs that are too hydrophobic and agglomerate easily, while low substitution is insufficient to drive self-assembly [26]. In the current study, we applied this method to graft CUR to the CS. PEG grafted to the amphiphilic CUR-grafted-CS (CCS) constitutes the hydrophilic part; however, its effects on self-assembly process and formation of the NPs’ physical and biological properties have not been studied. We are particularly interested in how the grafted density of PEG correlated with the characteristics and the biological functions of PCCM NPs. Thus, we prepared three of PCCM NPs with different PEG/CS feed ratio and evaluated the correlation between PEG/CS ratio and the NP characteristics, drug potency and the synergism of MTO and CUR in combination. Finally, the effect of pH on drug release from these three PCCM NPs was determined as a proxy for assessing the drug’s release under the physiological condition and the acidic microenvironment of cancer.

## 2. Materials and Methods

### 2.1. Materials

Chitosan (average molecular weight 20 kDa, degree of deacetylation > 90%) was purchased from Solarbio (Beijing, China). Curcumin, methoxy polyethylene glycol (2000 Da) and mitoxantrone hydrochloride were purchased from Sigma-Aldrich (Shanghai, China). 1-Ethyl-3-(3-dimethylaminopropyl) carbodiimide hydrochloride (EDCI) and 4-dimethylaminopryidine (DMAP) were purchased from Aladdin Reagent Co. Ltd. (Shanghai, China). Dulbecco’s modified Eagle Medium (DMEM) was purchased from Gibco (Grand Island, NY, USA). Hepg2 cell line was from Shanghai Institutes for Biological Sciences, Chinese Academy of Sciences (Shanghai, China).

### 2.2. Synthesis of Polyethylene Glycolated (PEGylated) Curcumin-Grafted-Chitosan (PCC) Conjugates

**Figure 1:**
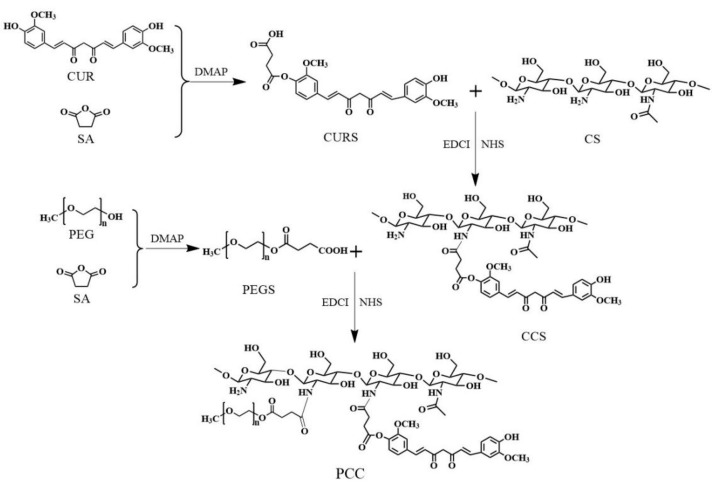
The chemical synthesis of Polyethylene glycolated (PEGylated)curcumin-grafted-chitosan (PCC).

The synthetic process of CUR-grafted-CS (CCS) and PCC are illustrated in Figure 1.

Synthesis of CCS: First, CUR succinate (CURS) was synthesized based on a previously published method with slight modifications [27]. Next, CCS conjugate was prepared by chemically grafting CURS to CS through amide formation. In brief, 0.065 g succinic anhydride (SA, 0.65 mmol) and 0.08 g DMAP (0.65 mmol) were dissolved in 15 mL dimethyl sulfoxide (DMSO); after heating and stirring at 50 °C for 4 h, 0.2 g CUR (0.54 mmol) was added for heating and stirring for 24 h under N_2_ protection to obtain CS. Subsequently, 0.12 g EDCI (0.62 mmol) and 0.075 g NHS (0.65 mmol) were added to the DMSO solution containing the CURS, and stirring was continued at 25 °C for 12 h. At the same time, 0.35 g CS (2.16 mmol) was dissolved in acetate buffer (200 mM, pH 5.0) to get a 0.5% (*w*/*v*) solution. Lastly, the CS solution was slowly added to the CURS solution, and these were reacted under N_2_ at 25 °C for 48 h. After the reaction, the resulting suspension was added into 200 mL absolute ethanol. The precipitate formed and went through vacuum filtration, then washed with absolute ethanol, tetrahydrofuran and diethyl ether, respectively, and dried to yield CCS conjugate.

Synthesis of PCC: PEG succinate (PEGS) was synthesized as described in [28]. In brief, 0.15 g SA (1.5 mmol) and 0.18 g DMAP (1.5 mmol) were dissolved in 15 mL dry CH_2_Cl_2_; after heating at reflux for 3 h at 60 °C, 2 g PEG (1.0 mmol) was added for heating and stirring for 24 h. The resulting solution was precipitated by diethyl ether and then filtered. The white powder was dissolved in deionized water after drying, then dialyzed against water for 2 days and the solution was lyophilized. PCC was produced by conjugating the carboxylic acid group of PEGS with the amine group of CS at PEG/CS molar ratios of 1/5, 1/7.5, and 1/10 (PCC1, PCC2, and PCC3) in the presence of EDCI/NHS. Then, 0.2 g PEGS (0.096 mmol), 0.014 g NHS (0.12 mmol) and 0.022 g EDCI (0.012 mmol) were dissolved in 10 mL DMSO and stirred at 25 °C for 4 h. At the same time, CCS (0.08 g, 1/5; 0.12 g, 1/7.5; 0.16 g, 1/10) was dissolved in acetate buffer (200 mM, pH 5.0) to get a 0.5% (*w*/*v*) solution. Lastly, the CCS solution was slowly added to the PEGS solution, and these were reacted under N_2_ at 25 °C for 48 h. The resulting suspension was washed with CH_2_Cl_2_/methanol (volume ratio: 4:1) and centrifugated at 8500 rpm for 10 min, the supernatant was removed. The above process was repeated three times and the precipitate was rinsed with deionized water and lyophilized to obtain three of PCC conjugates.

### 2.3. Fourier Transform Infrared (FTIR), Proton Nuclear Magnetic Resonance (^1^H NMR) and Ultraviolet (UV-Vis) Spectroscopy

The FTIR spectra for CUR, CS, CCS, PEG and PCC1 were recorded on FTIR spectrometer (Nicolet, TM Nexus 470-ESP, Thermo Fisher Scientific, Waltham, MA, USA) using KBr pellets. The ^1^H NMR spectra for CUR and PEG were recorded on a 500 MHz NMR spectrometer (BRUKER AVANCE-500, Bruker, Billerica, MA, USA) using DMSO-d6 solvent. The ^1^H NMR spectra for CS, PCC1, PCC2 and PCC3 were recorded on the same NMR spectrometer using CD_3_COOD/D_2_O solvent (1%, *v*/*v*). CUR was dissolved in methanol to obtain 200 µg/mL solution and PCC conjugates and CS were dissolved in acetic acid (1%, *v*/*v*) to obtain 1 mg/mL (*w*/*v*) for each solution. Then, the absorbances of these solutions were scanned from 200 nm to 700 nm wavelengths using an UV-Visible spectrophotometer (Shimadzu UV-2550, Kyoto, Japan).

### 2.4. Preparation PCC Nanoparticles (NPs)

PCC NPs were prepared by dialysis method [25]. Briefly, 5 mg PCC was suspended in 10 mL of 1% acetic acid solution under gentle shaking at 37 °C until it was completely dissolved and then dialyzed against 2000 mL of distilled water for 24 h with 10 exchanges by using a dialysis bag (molecular weight cut-off 8000–14,000 Da) to remove acetic acid. Then, the solution was sonicated using a probe type sonifier at 100 W with pulsing (pulse on 2.0 s, off 2.0 s) for 2 min in an ice water bath. The self-assembled PCC NPs were then filtrated through 0.45 µm-membrane and stored at 4 °C.

### 2.5. Dynamic Light Scattering (DLS) and Transmission Electron Microscopy (TEM)

The size and zeta-potential of different PCC NPs were determined by DLS (Zetasizer 3000 HS, Malvern Instruments, Malvern, UK). The NPs suspensions were filtered with a 0.45 μm filter, and each batch was analyzed in triplicate. To observe the morphologic features of PCC NPs, one drop of PCC NPs suspension was placed on carbon-coated 300 mesh grids. Then, the grids were air-dried and examined by TEM (Tecnai G2 20 S-Twin, FEI Hong Kong Inc., Hong Kong, China) at an accelerating voltage of 80 KV.

### 2.6. Preparation and Characterization of Mitoxantrone-loaded PCC NPs (PCCM NPs)

PCC conjugate (20 mg) was suspended in 20 mL of 1% acetic acid solution under gentle shaking at 37 °C until it was completely dissolved. Then, 2 mg MTO was dissolved in 2 mL of DMSO, dropped into the above solutions, dialyzed for 24 h in distilled water using a dialysis bag (molecular weight cut-off 8000–14,000 Da) to remove organic solvent and free MTO, and the PCCM NPs solution was obtained. MTO-loaded PCC1, PCC2 and PCC3 NPs were obtained in the same way and named PCCM1, PCCM2 and PCCM3 respectively.

### 2.7. Determination of Entrapment Efficiency (EE) and Loading Capacity (LC)

PCCM NPs solution (5 mL) was sonicated for 5 min (pulse on 2.0 s, off 2.0 s) to release the drug from NPs. The absorbances of MTO and CUR in the solution were measured at 608 nm and 425 nm, respectively, by microplate spectrophotometer (UV-384 plus, Molecular Devices, Thermo Fisher Scientific Inc., Waltham, MA, USA) to calculate the drug concentrations. MTO encapsulation efficiency (EE), MTO load capacity (LC_M_) and CUR load capacity (LC_C_) were calculated as follows:

EE% = (the amount of drug in the nanoparticles)/(the amount of totally added drug) × 100%

LC% = (the amount of drug in the nanoparticles)/(the amount of nanoparticles weight) × 100%

### 2.8. Determination of Drug Release from PCCM NPs In Vitro

The effect of pH on drug release was measured by a dialysis method as previously described [24]. Briefly, PCCM NPs solution (5 mL) was put into a dialysis bag (8–12 kDa MWCO) and dialyzed in 25 mL of PBS (releasing media) with pH 7.4, 6.8 or 4.0 at 37 °C under 100 rpm shaking. Free MTO was dialyzed under the same conditions. Then, 2 mL of releasing medium was collected for sampling and replaced with an equal volume of the fresh solution at pre-defined time intervals (*T_n_*, *n* = 0, 0.5, 1, 2, 4, 8, 12, 24 and 48 h). The absorbances of MTO and CUR in the solution were measured by microplate spectrophotometer to determine the concentrations of MTO and CUR released. The percentage rate of drug release (*Q*%) was calculated as follows:

$$Q\%=\left(C_{n}\times V+V_{n}\overset{n}{\underset{t=0}{\sum }}C_{i}\right)/\left(W_{NP}\times LC\%\right)$$

where *W* is NPs weight; *C_n_* is the sample concentration at *T_n_*; V is the total volume of release medium; *V_n_* is the sample volume (2 mL); and *C_i_* is the sample concentration at *T_i_*(*i* = 0, 0.5, 1,…,*n* h, both *V_0_* and *C_0_* are equal to zero).

### 2.9. Determine PCCM NPs’ Cytotoxicity In Vitro

MTT assay was used to determine the drug cytotoxicity by measuring HepG2 cell viability. HepG2 was cultured in DMEM medium supplemented with 10% FBS and 100 U of penicillin–streptomycin in a humidified atmosphere of 95% air and 5% CO_2_ incubator at 37 °C. HepG2 cells were seeded at 20,000 cells/well in 96-well plates and incubated overnight. Then, the drug was added and incubated for 24 h. The medium was removed and started MTT assay to determine cell viability. MTT assay was performed according to the manufacturer’s protocol. The percentage of cell viability was calculated based on the ratio of the absorbance of drug-treated cells to that of untreated cells. The untreated cell was counted as 100% survival. The dose–effect curves of HepG2 cell viabilities were performed under the treatments of PCCM1, PCCM2 and PCCM3. The MTO concentration in PCCM NPs was determined by the absorbance at 608 nm wavelength and the MTO treatment concentration was fixed at 2, 4, 8, 16 and 32 µg/mL for each PCCM. Because the PCCM NPs contained MTO and CUR, and the loading capacity of CUR and MTO has been determined (Table 1), the CUR concentrations in PCCM NPs were estimated based on the ratio of MTO/CUR loading capacity (Table 2). The PCCM treatment concentration was the sum of MTO and CUR.

To determine the synergistic effect of MTO and CUR in combination, the dose–effect curves with the individual treatment of free MTO (2, 4, 8, 16, 32 and 64 µg/mL), free CUR (2, 4, 8, 16, 32 and 64 µg/mL) and the combination of both with MTO:CUR ratios at 1:1, 1:1.5 and 1:2 against HepG2 cell viability were performed. In drug preparations, DMSO was used to dissolve free MTO and free CUR to make each stock solution of 32 mg/mL that was further diluted with DMEM medium to achieve the desired concentrations. The percentage of cell viability was calculated based on the ratio of the absorbance of drug-treated cells to that of cells treated equal volume of DMSO. The maximal concentration of DMSO in the experimental medium was less than 0.5% (*v*/*v*), which did not affect cell viability.

### 2.10. Calculation of the Synergistic Effect of MTO and CUR

According to the Chou-Talalay model [29,30], the combination index (*CI*) was computed based on the following equation:

$$CI=\left[\frac{p}{\left(p+q\right)EC_{50,\;\mathrm{MTO}}}+\frac{q}{\left(p+q\right)EC_{50,\;\mathrm{CUR}}}\right]EC_{50,\;combination}$$

where *p* and *q* represent the unit of drug MTO and CUR, respectively. *EC_50_* denotes the drug dose at 50% of cell viability achieved. If *CI*<1, the combination can be described as synergistic, if *CI* > 1, antagonistic, and *CI* = 1, additive. The data for calculating *CI* are presented in Table S1.

Isobole analysis is another way to quantitatively assess the synergism and antagonism that paired drugs produce. According to Tallarida’s dose equivalent principle and Loewe additive model, an isobole is generated, which is a line to define the additive effect of paired drugs [31,32]. In practice, we first acquired the dose-effect curves of free MTO and free CUR and transformed the drug dose in log_10_ scale, and then applied the following equation to calculate the combined doses of the paired drugs to give a specified effect.

$$\ln\frac{P_{x}}{1\;-\;P_{x}}=\alpha +\beta x$$

where *x* is the log_10_ dose of a drug (MTO or CUR); *P_x_* is cell viability at log_10_ dose;(1 − *P_x_*) is the cell death at log_10_ dose; α is the Y-intercept of linear regression equation and β is the slope. The data for plotting the isobole are illustrated in Table S2. The points on the isobole set the combined CUR and MTO at different ratios to produce a 50% of maximum effect. If the EC_50_ of the paired drug dose is located below the isobole, it indicates a synergistic effect, whereas, above the isobole indicates an antagonistic effect.

### 2.11. Statistical Analysis

All experiments were performed at least three times in vitro. Results are expressed as mean ± standard deviation (SD), analyzed by Student’s *t*-test using software SPSS 19.0. *p* < 0.05 was considered significantly different.

## 3. Results

### 3.1. FTIR, ^1^H NMR and UV-Vis Spectroscopic Analysis

**Figure 2:**
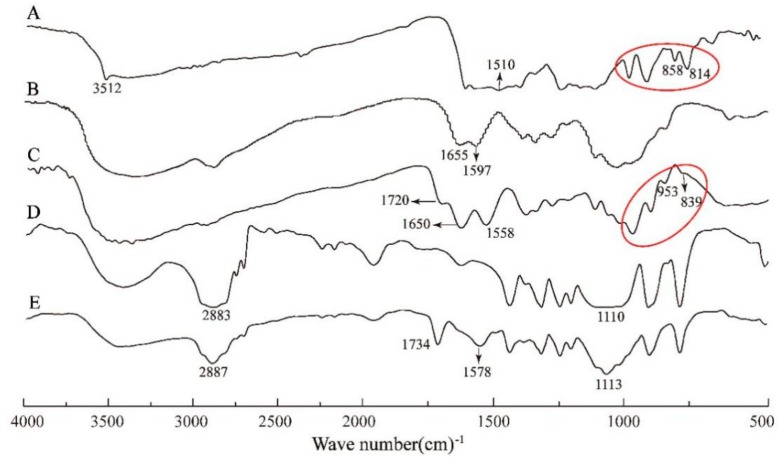
Fourier transform infrared (FTIR) spectra of: Curcumin (CUR) (**A**); Chitosan (CS) (**B**); CUR-grafted-CS (CCS) (**C**); polyethylene glycol (PEG) (**D**); and PEGylated CUR-grafted-CS (PCC) (**E**).

The successful syntheses of these compounds were confirmed by FTIR spectra, as shown in Figure 2. In comparison with CUR (A) and CS (B), the spectrum of CCS (C) remained the N-H bond of amino at 1558 cm^−1^ and the aromatic C=C bonds of CUR (marked with red circle). The spectrum of CCS also revealed the particular peak of amide bond at 1650 cm^−1^ (C=O stretching), and the peak of ester bond at 1720 cm^−1^ (C=O stretching). This result indicated the successful conjugation between CUR and CS. In the spectrum of PCC, peaks at 2887 cm^−1^ (C−H stretching) and 1113 cm^−1^ (C−O stretching) corresponded to the characteristic peaks of PEG. The peak of ester bond was shifted to 1734 cm^−1^. The FTIR spectra confirmed the chemical structure of PCC.

**Figure 3:**
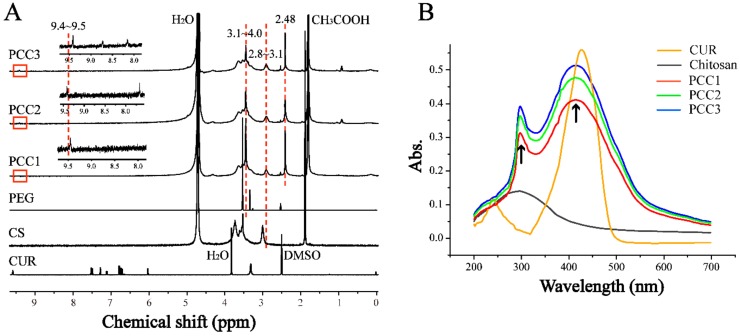
(**A**) Nuclear Magnetic Resonance (^1^H NMR) spectra for CUR, CS, PEG, PCC1, PCC2 and PCC3; and (**B**) Ultraviolet (UV-Vis) spectra of CUR, CS, PCC1, PCC2 and PCC3.

Figure 3A displayed the ^1^H NMR spectra of CUR, CS, PEG, PCC1, PCC2 and PCC3. The characteristic peaks 2.8 to 3.1 ppm were assigned to the monosaccharide residue (CH-NH-) protons. In the spectrum of PCC conjugates, the highly enhanced peaks at 3.1~4.0 ppm corresponded to the repeated ethyl group (−CH_2_−CH_2_−O−). The peaks at 2.48 ppm corresponded to the methylene (CH_2_) protons in succinate linkers between PEG and CS and between CUR and CS. The peaks at 9.4~9.5 ppm were characteristic peaks of CUR, the other characteristic peaks of CUR were not observed, which might be because PCC conjugates’ MW were too high [33]. The results of ^1^H NMR spectra confirmed the successful synthesis of PCC conjugates. The degree of substitution (DS) of PEG residues per 100 sugar units for CS could be calculated by the ratio between the increased integrity at 3.1−4.0 ppm and the monosaccharide residue (CH−NH−, 2.8−3.1 ppm) and further adjusted with PEG molecular weight to obtain molar substitution degree [28,34]. The DS of obtained PCC1, PCC2 and PCC3 were determined as 0.75%, 0.45% and 0.33%, respectively. The UV-Vis spectra were further used to confirm CUR in PCC conjugates’ structure. We used the same concentration in each PCC (1 mg/mL) and scanned the PCC absorbance from 200 nm to 700 nm wavelength. As depicted in Figure 3B, the characteristic peaks at 297 nm and 415 nm belonged to chitosan and CUR, respectively. The absorbances of PCC conjugates displayed the following order: PCC3 > PCC2 > PCC1. Therefore, the UV-Vis spectra demonstrated the presence of CUR and CS in the conjugates. PCC3 was composed of more CUR and CS as compared with PCC1 and PCC2 with the same weight.

### 3.2. Size Distribution, Zeta Potential and Morphology of PCC NPs

**Figure 4:**
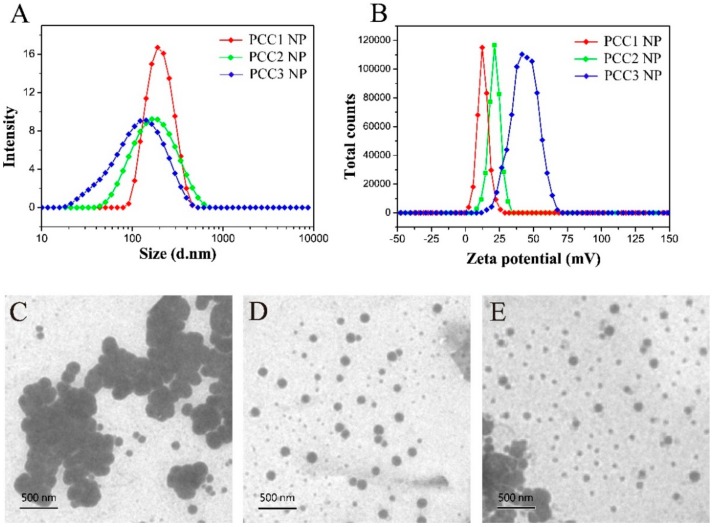
The size distributions (**A**); and zeta potential (**B**) of PCC1, PCC2 and PCC3 NPs. Transmission electron microscopy (TEM) images of: PCC1 NP (**C**); PCC2 NP (**D**); and PCC3 NP (**E**).

The mean distribution size and polydispersity index (PDI) were, respectively,209.9 nm and 0.076 for PCC1 NP; 177.6 nm and 0.247 for PCC2 NP; and 137.4 nm and 0.267 for PCC3 NP (Figure 4A). The zeta potentials of PCC1, PCC2 and PCC3 NPs were 12.9 ± 4.02 mV, 21.3 ± 4.27 mV and 34.4 ± 9.52 mV, respectively (Figure 4B). As the ratio of PEG/CS decreased, the size of PCC decreased and the zeta potential increased. TEM images show the sphere shape of PCC NPs (Figure 4C–E).

### 3.3. Characterizations of PCCM NPs

**Figure 5:**
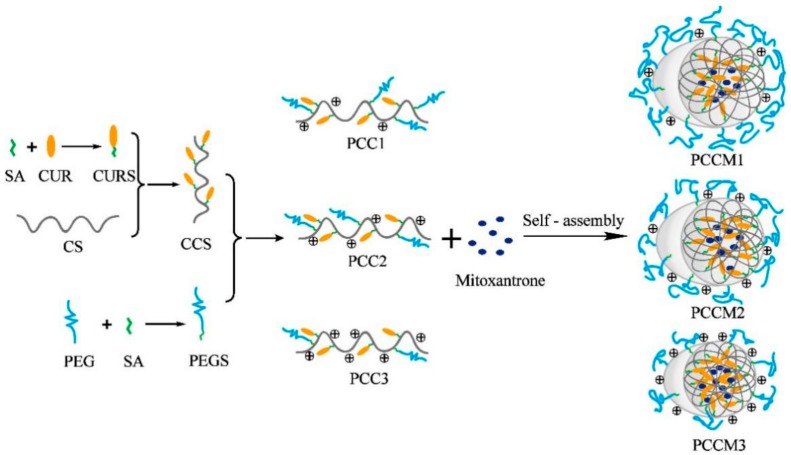
Schematic diagram illustrated the design and self-assembly of PCCM NPs with different density of PEG in each of PCCM.

Figure 5 schematically illustrates the formation of PCCM NP. As denoted in our design, the hydrophobic molecule CUR was conjugated to the CS polymer and formed the hydrophobic core in the self-assembly process. On the external shell, we varied the ratio of PEG/CS to make three kinds of PCCM NPs and evaluated the properties and biological functions of the NP due to the change of PEG/CS ratio. Characterizations of all of PCCM NPs confirmed our designs, as summarized in Table 1. The PEG/CS ratio in the composition of the external shell significantly affected NPs’ properties. Higher degree of PEG grafted on the external shell rendered alarger size distribution, less positive zeta potential and lower drug loading capacity of the NPs (e.g., PCCM1). As the PEG/CS ratio was reduced, all changes of the NPs properties were reversed accordingly (e.g., PCCM2 and PCCM3). The change in PCCM3 was remarkably optimal. It is reasonable to account for the correlations of the PEG/CS and the NPs’ characterizations regarding the NPs size, zeta potential and drug loading capacity. The PEG grafted to amino group of the CS could mask the positive charge. More PEG molecules grafted to the CS backbone reduced the area of positively charged CS exposed, which resulted in lowering the zeta potential, or vice versa (Figure 5). In aqueous solution the amphiphilic PCC conjugate formed the micelle with the PEGylated CS hydrophilic chain facing externally. Solvents can mediate aggregation of NPs by H-bonding [22,24]. PCCM with low PEG/CS ratio corresponding to highly positive charges would have more H-bonding connections and result in forming highly compact NPs. As expected, PCCM3 formed in the smallest size, the largest positive zeta potential, but with the highest drug loading capacity. Particularly, the PEG/CS ratio had significant impact on the loading capacity for CUR than that for MTO (Table 1). This was because the assembly of conjugated CUR was directly correlated to the degree of PEG. Since MTO and CUR are hydrophobic molecules, larger amounts of assembled CUR would associate with more MTO being encapsulated. The loading capacities for MTO were indirectly associated with the PEG/CS ratio.

### 3.4. Mitoxantrone (MTO) and Curcumin (CUR) Drug Release from PCCM NPs In Vitro

**Figure 6:**
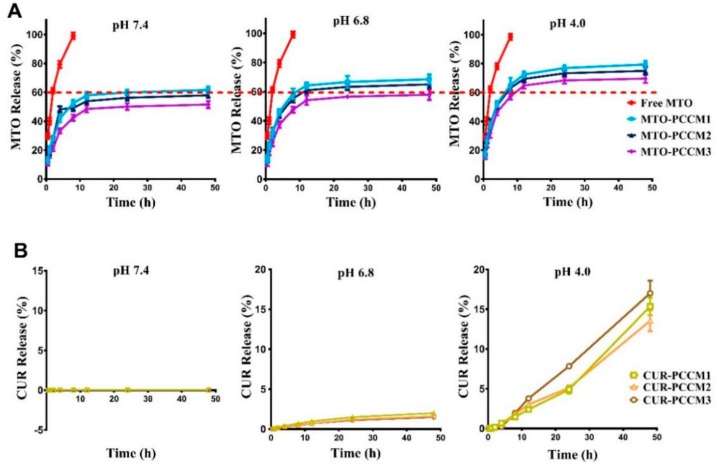
In vitro release profiles of Mitoxantrone (MTO) (**A**) and CUR (CUR) (**B**) from PCCM NPs in pH 7.4, 6.8 and 4.0 conditional media.

Figure 6 shows the MTO (Figure 6A) and CUR (Figure 6B) release from PCCM NPs at pH 7.4, pH 6.8 and pH 4.0 conditional media. All PCCM NPs exhibited two phases of MTO releasing profile, a rapid release in 10 h followed by a sustained release in 48 h. Free MTO completely released within 8 h under the same conditions. The rapid releasing MTO in the first 10 h probably related to the surface-absorbed MTO [35]. The encapsulated MTO sustained release slowly. Larger sizes of NPs or lower pH conditions were associated with higher percentage of MTO release, displayed in the following order: PCCM1>PCCM2>PCCM3, and the release at pH 4.0 > pH 6.8 > pH 7.4 (Figure 6A). At pH 7.4 conditional medium that mimicked extracellular circulation condition, the MTO release from PCCM3 NP was about 51.54%, which was significantly lower than that from PCCM2 (57.96%) and PCCM1 (61.64%). We anticipated PCCM3 would have the least MTO loss in systemic circulation.

The release of CUR could only be detected at pH 4.0 (Figure 6B). The cumulative release of CUR for 48 h from PCCM1, PCCM2 and PCCM3 was 15.36%, 13.55% and 17.02%, respectively. At pH 6.8 and pH 7.4, the CUR release was close to zero. Therefore, we expect CUR loss was zero in physiological circulation system as long as the NPs were intact. CUR release required breaking the chemical bond between CUR and CS and was exclusively pH dependent, as the CUR released only at pH 4.0 in a time dependent manner. Under the acidic condition such as the microenvironment of cancer cells, acid catalyzed the hydrolysis of amide bonding between CS and CUR and the free CUR gradually diffused out of the NPs. The order of the percentage of CUR release in PCCM NPs was: PCCM3 > PCCM1 > PCCM2. The relatively high level of CUR release in PCCM3 might be related to its larger amount of CUR loaded.

### 3.5. Cytotoxicity Test In Vitro

**Figure 7:**
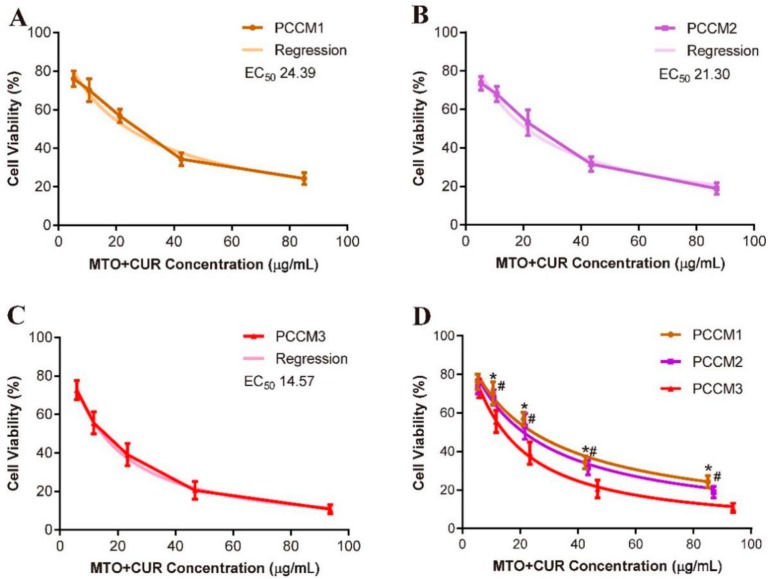
Dose–effect curves and the regressions of: PCCM1 (**A**); PCCM2 (**B**); PCCM3 (**C**); and all PCCM NPs for comparison (**D**). Data represent means ± SD (*n* = 6). Statistics: * indicates significant differences between PCCM1 and PCCM3 (*p* < 0.05); # indicates significant differences between PCCM2 and PCCM3 (*p* < 0.05). There was no significant difference between PCCM1 and PCCM2.

The treatments of PCCM NPs greatly reduced HepG2 cell viability (Figure 7). PCCM3 displayed the highest level of cytotoxicity while exhibiting the lowest EC_50_ value (14.57 ± 0.78 µg/mL), and the lowest percentage of cell viability (10.82% ± 2.32%) as compared to that of PCCM1 and PCCM2. The biological effects of drugs were dose dependent [36] and correlated with the efficiency of drug endocytosis [37] and, perhaps, the synergism of the drugs in combination [3,38]. We administered uniform MTO concentrations across all PCCM NP treatment groups, but varied the CUR concentrations (Table 2). Among PCCM NPs tested, PCCM3 obtained the highest proportion of CUR (MTO:CUR, 1:1.925), and may partially account for the significantly enhanced cytotoxic effect of PCCM3, as CUR is known to mediate synergistic effects when co-administered with some chemotherapeutic drugs. Moreover, the positively charged of NPs can promote cellular uptake through electrostatic interaction between NPs and cell membrane leading to NPs endocytosis [17,39]. The high positively charged surface of PCCM3 may facilitate NPs endocytosis.

**Figure 8:**
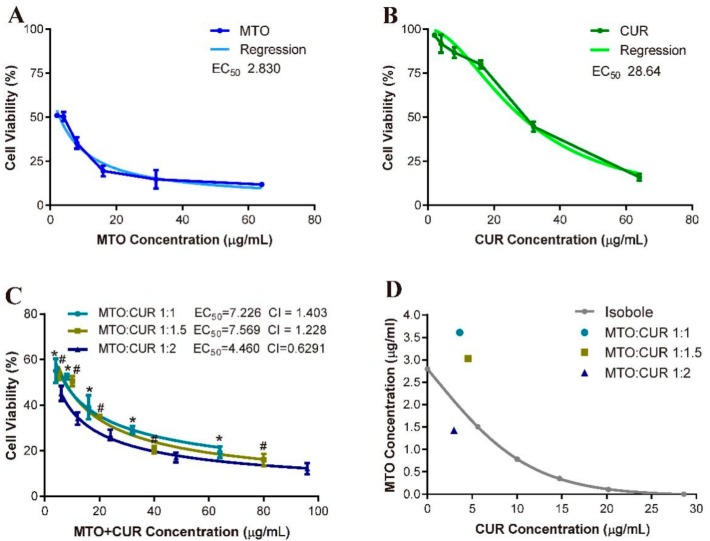
Dose–effect curves and regressions of: free MTO (**A**); and free CUR (**B**); the combination of MTO and CUR at the combination ratio of 1:1, 1:1.5 and 1:2 (**C**); and the isobole (**D**). Data represent means ± SD (*n* = 6). Statistics: * indicates significant differences between the models of MTO:CUR at 1:1 and 1:2 (*p* < 0.05); # indicates significant differences between the models of MTO:CUR at 1:1.5 and 1:2 (*p* < 0.05).

Next, we addressed whether the combination of CUR with MTO could mediate synergistic effect, and, if so, whichr atios of MTO and CUR would achieve the synergism. We applied the mass-action law model proposed by Chou-Talalay to compute the combination index (*CI*), where *CI* < 1 indicates paired drug effects of synergism; *CI* = 1 indicates additivity; and *CI* > 1 indicates antagonism [30,31]. We generated dose–effect curves using free MTO and free CUR (without NPs encapsulation) to determine the *CI* values of MTO:CUR at 1:1, 1:1.5 and 1:2 (Figure 8A–C). *CI* < 1 was only observed when the MTO:CUR was at 1:2 (CI_MTO:CUR 1:2_ = 0.629), whereas, *CIs* > 1 were observed at the other combination ratios (CI_MTO:CUR 1:1_ = 1.403, CI_MTO:CUR 1:1.5_ = 1.228). A combination ratio of MTO and CUR at 1:2 was critical, at which the synergism was likely achieved. We also used isoboles method to quantitatively assess the synergism and antagonism of the paired drug effects [33]. Based on the doses of MTO and CUR with different combination ratios to achieve 50% of maximum effect, we plotted an isobole that indicated the additive effect of the paired drugs, which allowed us to define the area of super additivity (synergism) or subadditivity (antagonism). Again, only the EC_50_ dose of MTO and CUR at 1:2 combination model was localized below the isobole, suggesting combined MTO and CUR at this ratio would likely produce synergistic effect (Figure 8D). The rationale of the free drug models could be applied to explain the observed results of PCCM NPs. The EC_50_ values of PCCM1, PCCM2 and PCCM3 were 24.39, 21.15 and 14.57 µg/mL and corresponded to the MTO and CUR combination ratios of 1:1.66, 1:1.72 to 1:1.925, respectively. Lower *EC_50_* indicates higher the drug potency. The significant increase in the potency of PCCM3 compared to other PCCMs suggested that the MTO/CUR combination ratio in PCCM3 played a critical role in enhancing the drug combination effect toward super additivity.

## 4. Discussion

CUR has been widely considered an adjuvant drug for chemotherapy [3,40,41]. Many studies have reported about the combination of CUR with chemotherapeutic drugs that have demonstrated the enhancement of antitumor effect by CUR and nanonization of CUR that can improve its bioavailability [41,42,43]. By these methods, CUR was adsorbed on surface of nanoparticles or encapsulated in core of liposomes or micelles. The use of chemically grafting CUR to CS is a novel design that incorporates CUR into NPs structure and allows CUR to be carried. This method offers several advantages: first, the quantity of CUR in the nanocarrier can be regulated because the conjugation of CUR molecules to the CS polymer is a chemical reaction that follows the reaction stoichiometry principle. Once the quantity of CUR is fixed, the proportional MTO molecules associated with CUR by hydrophobic interaction are encapsulated. Secondly, the free drug dose–effect models (Figure 8) suggested that a high proportion of CUR was prerequisite to achieve the synergistic effect in combination with MTO. Relatively, MTO is a highly potent chemotherapeutic drug with adverse cytotoxic side effects and should be kept in low concentration. The design of PCCM NPs is desirable to model CUR and MTO in combination. Using two different approaches to encapsulate two drugs, we can manipulate the drug ratio and ultimately maximize the drug efficacy and the synergism. Thirdly, unloaded CUR required breaking the chemical bonding in acidic conditions. Thus, CUR delivery could be targeted to the tumor cells where the acidic microenvironment was favorable for CUR release.

The strategy of grafting PEG on the NPs surface to escape RES phagocytosis is a sophisticated method [44]. Recently, Yang et al. demonstrated the impact of PEGylation on characterization of CS NPs properties, bioactivities and tissue distributions in vitro and in vivo [20]. Our study used similar methodology for PEGylation and both PEGylated-chitosan NPs displayed comparable results in terms of sizes, zeta potentials and PEG content. Yang’s study reported that higher molecular weight and lower grafting rates of PEG resulted in forming smaller and more compact NPs with relatively higher surface charge, which was consistent with our findings. Yang et al. further revealed that PEGylated-CS NPs significantly inhibited macrophage phagocytosis and unspecific interaction with red blood cells. Gref et al. [45] also described that, in PEG concentration above 5%, PEG functioned as a “brush” which effectively shielded the surface charge of the nanoparticles, thereby prolonging the retention time of NPs in circulation and stabilizing NPs structure in vivo. These findings suggest that the PEGylated-CS NPs developed in the current study could be applicable for systemic circulation.

It is well known that synergism or antagonism in a drug pair depends not only on the agonist drug pair, but also on the ratio of the doses. Often, there is a range of dose combinations that are synergistic and other ranges that are either additive or antagonistic [32]. We validated that MTO:CUR at 1:2 is the critical combination ratio to achieve synergistic effect based on the *CI* values and the method of isobolo gram (Figure 8). Most importantly, in this study, we elucidated the rationale of grafted PEG density in the PEGylated CS NPs resulting to alter the drug potency and synergism, and finally presented PCCM3 as the best nanocarrier model for CUR and MTO. PCCM3 with the lowest PEG density (PEG/CS 1:10) and the optimal combination ratio of MTO and CUR that close to 1:2 has demonstrated the best characteristics and the anticancer effects among the three PCCM NPs examined.

## 5. Conclusions

We successfully fabricated a new design of a drug carrier for MTO and CUR by chemically linked CUR and physically loaded MTO. In evaluating three of PCCM NPs that consisted of different PEG/CS ratio, PCCM NPs with the smallest DS of PEG were determined as the best with regard to their physical properties and the better anticancer effect. Our results established the relationship between physical properties and the biological functions of PCC NPs. We further explained a proper combination ratio of MTO and CUR would achieve synergistic cytotoxicity to cancer cells. Our findings provide new insights in CUR drug carrier development, particularly CUR in combination with the chemotherapeutic drugs for maximizing synergistic effects, thus exhibiting great potential for applications in the combination of CUR and chemotherapeutic drugs for cancer therapy.

## Figures and Tables

**Table 1:** Characterization of mitoxantrone-loaded PCC NPs (PCCM NPs).

Sample	Feed PEG/CS Molar Ratio	PEG Molar DS	EE%	LC_M_%	LC_C_%	Zeta Potential (mV)	Size (nm)	PDI
**PCCM1**	1/5	0.75%	90.5 ± 2.89	7.42 ± 0.16 *,#	12.3 ± 0.52 *,#	12.8 ± 4.02 *,#	250.2 ± 21.5 *	0.153
**PCCM2**	1/7.5	0.45%	88.6 ± 1.61	8.14 ± 0.14	14.0 ± 0.87 *	21.2 ± 4.27 *	233.1 ± 19.2 *	0.216
**PCCM3**	1/10	0.33%	87.3 ± 1.74	8.30 ± 0.24	16.1 ± 0.21	34.0 ± 4.52	183.1 ± 15.6	0.225

Data were expressed as mean ± standard deviation (SD) (*n* = 3). * indicates the significant difference of PCCM1 and PCCM2 vs. PCCM3 (*p* < 0.05); # indicates the significant difference between PCCM1 and PCCM2 (*p* < 0.05). EE%, encapsulation efficiency of MTO; LC_C_%, loading capacity of CUR; LC_M_%, loading capacity of mitoxantrone (MTO); polydispersity index (PDI).

**Table 2:** The concentrations of mitoxantrone (MTO) and chemotherapy (CUR) in PCCM NPs on the treatments of HepG2 cells.

Drugs	Concentration (μg/mL)				
**PCCM NPs-MTO**	2.00	4.00	8.00	16.00	32.00
**PCCM1-CUR**	3.32	6.63	13.26	26.52	53.05
**PCCM2-CUR**	3.44	6.88	13.70	27.50	55.00
**PCCM3-CUR**	3.85	7.71	15.40	30.80	61.70

## Supplementary Materials

The following are available online at http://www.mdpi.com/2079-4991/8/7/486/s1, Table S1: Supplemental data for calculating the combination index, Table S2: Data for plotting the isobole.
