# Supplementary material for: Evaluation of the PEG Density in the PEGylated Chitosan Nanoparticles as a Drug Carrier for Curcumin and Mitoxantrone
Source: Nanomaterials (Basel). 2018 Jul 1;8(7):486. doi: 10.3390/nano8070486 (PMC6071138; doi:10.3390/nano8070486)
Supplement: Supplementary file 1 [file nanomaterials-08-00486-s001.pdf]

nanomaterials

MDPI

Supplementary Materials

Article

Evaluation of the PEG Density in the PEGylated Chitosan Nanoparticles as a Drug Carrier for Curcumin and Mitoxantrone

Yao Chen, Di Wu, Wu Zhong, Shu Kuang, QianLuo, Liujiang Song, Lihua He, Xing Feng and Xiaojun Tao *

Key Laboratory of Study and Discovery of Small Targeted Molecules of Hunan Province, School of Medicine, Hunan Normal University, Changsha 410013, China; ychen@smail.hunnu.edu.cn (Y.C.); iswudi@smail.hunnu.edu.cn (D.W.); zwu123@smail.hunnu.edu.cn (W.Z.); shwkuang@smail.hunnu.edu.cn (S.K.) luoqian1996@smail.hunnu.edu.cn (Q.L.); song1221@hunnu.edu.cn (L.S.); hlh197@hunnu.edu.cn (L.H.); fengxing@hunnu.edu.cn (X.F.)

* Correspondence: xjtao@hunnu.edu.cn (X.T.); Tel.: +86-182-2972-2319 (X.T.)

Received: 15 May 2018; Accepted: 8 June 2018; Published: date

Table S1. Supplemental data for calculating the combination index

Sample	P(MTO)/Q(CUR)	EC_{50} (μg/mL)	R square	CI
Free MTO	-	2.830	0.922	-
Free CUR	-	28.64	0.976	-
Combination	1/1	7.226	0.948	1.403
Combination	1/1.5	7.569	0.937	1.228
Combination	1/2	4.460	0.963	0.6291
PCCM1	1/1.66	24.39	0.952	-
PCCM2	1/1.72	21.30	0.948	-
PCCM3	1/1.925	14.57	0.968	-

Table S2. Data for plotting the isobole

**CUR**			**MTO**			**Addition effect $(P_x)_C+(P_x)_M$**	**Addition dose $(Dose_C+Dose_M)$ (μg/mL)**	**Dose pair $(Dose_C,Dose_M)$ (μg/mL)**
$\ln\frac{P_x}{1-P_x}=4.613-3.223x$			$\ln\frac{P_x}{1-P_x}=0.682-1.552x$					
$(1-P_x)_C$	X_C	$Dose_C$ (μg/mL)	$(1-P_x)_M$	X_M	$Dose_M$ (μg/mL)			
0%	-	0	50%	0.4393	2.750	50%	2.750	(0,2.750)
10%	0.7495	5.617	40%	0.1781	1.507	50%	7.124	(5.617,1.507)
20%	1.001	10.03	30%	-0.1065	0.7825	50%	10.81	(10.03,0.7825)
30%	1.168	14.74	20%	-0.4538	0.3517	50%	15.09	(14.74,0.3517)
40%	1.305	20.20	10%	-0.9763	0.1056	50%	20.31	(20.20,0.1056)
50%	1.431	27.00	0%	-	0	50%	27.00	(27.00,0)

Notes: Where "x" is a drug log10 dose (CUR or MTO); "P_x" is cell viability at log10 dose; $(1-P_x)$ is the cell death at log10 dose.

Dose pairs ($Dose_C$ and $Dose_M$) that plotted as points on the isobole (Figure 7.2D) represented the combination doses to produce a total 50% of maximum effect. The slope and the intercept of the equation were derived from the dose-effect curves of free CUR and free MTO (Figure 7.2A,B).

© 2018 by the authors. Submitted for possible open access publication under the terms and conditions of the Creative Commons Attribution (CC BY) license (http://creativecommons.org/licenses/by/4.0/).
